# Use of transcriptomics and genomics to assess the effect of disinfectant exposure on the survival and resistance of *Escherichia coli* O157:H7, a human pathogen

**DOI:** 10.3389/fmicb.2024.1477683

**Published:** 2024-10-23

**Authors:** Miranda Kirchner, Javier Nunez-Garcia, Nicholas Duggett, Rebecca J. Gosling, Muna F. Anjum

**Affiliations:** Department of Bacteriology, Animal and Plant Health Agency, Addlestone, United Kingdom

**Keywords:** adaptation, disinfection, *Escherichia coli*, O157:H7, resistance

## Abstract

Disinfectants are essential for biosecurity, preventing the persistence and spread of zoonotic pathogens on farms and subsequent human infections. In this study, transcriptomics and genomics were utilised to assess the effect of disinfectant exposure on pathogenic *Escherichia coli*. The exposure of *E. coli* O157:H7 to sub-optimal concentrations of commonly used farm disinfectants elicited changes in both the transcriptome and genome. The transcriptomics identified upregulation of >300 genes and downregulation of >100 genes with functions, which included stress response, metabolism, transcription, transportation, membrane-associated and virulence genes. The phage shock protein (*psp*) operon was highly upregulated in response to a quaternary ammonium compound (QAC)-containing disinfectant, which has not previously been associated with a response to chemical stress. Disinfectant-adapted isolates generated by exposure to sub-lethal disinfectants levels demonstrated resistance to several common antibiotics and decreased sensitivity to biocides. Whole genome sequencing of the mutant strains indicated that they had acquired mutations in the genes associated with the upregulation of the multiple antibiotic resistance (MAR) efflux system (*lon* protease and *marR*) and topoisomerase genes (*gyrA* and *gyrB*). The disinfectant-adapted isolates also exhibited increased expression of transcription, respiration and several pH stress response genes localised in the “acid fitness island.” This study demonstrated that sub-optimal disinfectant concentrations allow *E. coli* O157:H7 to adapt and survive disinfection and develop antibiotic resistance. These changes could have implications for disease treatment and elimination on farms. Although *E. coli* O157:H7 and farm disinfectants were the focus of this study, we believe these findings are also applicable to other settings, including hospitals.

## Introduction

Disinfectants are antimicrobial agents that are widely used in homes, healthcare facilities and farm environments to reduce levels of bacterial and viral contamination. In hospitals and farms, they help prevent microbial cross-contamination, maintain biosecurity and reduce the incidence of healthcare-associated infections, especially during a disease outbreak. There are many disinfectants on the market with different active ingredients and different modes of action (McDonnell and Russell, 1999). The UK government (Department of Environment, Food and Rural Affairs) approves disinfectants for the control of animal diseases. Disinfectants are approved at different concentrations for different functions, including General Orders (GOs) for routine cleaning and disinfection of farms.^1^ Protocols for infection control are also implemented in hospitals and public areas, with local authorities and other delegated authorities providing guidance. For example, the Centres for Disease Control and Prevention provides guidance regarding disinfection and sterilisation for infection control in the United States.^2^

Bacteria can become resistant to disinfectants through intrinsic mechanisms (McDonnell and Russell, 1999), such as the formation of disinfectant-resistant spores, the impermeable nature of Gram-negative cell membranes and the restriction of access to the cell cytoplasm. Bacteria can also acquire the mechanisms of disinfectant resistance via plasmids or integrons that encode disinfectant resistance genes, chromosomal mutations and the induction of efflux mechanisms to expel intracellular disinfectants. Several transporter families are associated with biocide and drug efflux pumps. These include the following: (1) ABC transporters; (2) resistance-nodulation-division (RND) transporters, such as multiple antibiotic resistance (MAR; Okusu et al., 1996); (3) the major facilitator superfamily (MFS), where *mdtM* can elicit quaternary ammonium compound (QAC) resistance when overexpressed in *E. coli*; (4) the multidrug and toxic compound extrusion (MATE) family; (5) staphylococcal multi-resistance (SMR) genes, such as *sugE*(c), *emrE* and *ydgE*/*ydgF*; and (6) the proteobacterial antimicrobial compound efflux (PACE) family, which also facilitates QAC resistance along with *qac* genes, which can be located on mobile genetic elements. For example, in *Staphylococcus*, the PACE family genes *qacA/B* and the *smr* gene can be co-localised on plasmids with other resistance determinants (antibiotic and heavy metals; Anes et al., 2015; Chapman, 2003). Several studies have demonstrated that resistance can be induced by the passage (growth) of bacterial pathogens, such as *Salmonella,* in sub-lethal concentrations of disinfectants, causing changes in the expression of efflux pumps from the wild-type phenotype (Condell et al., 2012; Whitehead et al., 2011; Nhung et al., 2015). This has also been demonstrated in *E. coli*, with mutations generated in several genes, including the efflux mechanisms *mdf* and *acr* (Merchel Piovesan Pereira et al., 2021).

The effect of environmental conditions, including the gut environment, on gene expression has been assessed in O157. It is known that stresses such as low-level chlorine or hypochlorite exposure can induce transcriptional changes in O157 (Wang et al., 2009). Gut metabolites (Liu et al., 2022) and the changes encountered in the gut during the transit of O157 through the digestive system are also known to affect the transcriptome of O157, including the upregulation of virulence gene expression (Feng et al., 2022; Nawrocki et al., 2020). Some studies have also assessed the effect of sub-lethal disinfectant exposure on the transcriptome of various bacterial species, including *E. coli* (Merchel Piovesan Pereira et al., 2020; Ligowska-Marzęta et al., 2019; Forbes et al., 2019).

*Escherichia coli* O157:H7, an enteric zoonotic pathogen, can cause human infections, resulting in nausea, vomiting, diarrhoea and sometimes fatal complications that can lead to death. In England and Wales, 563 human cases of STEC O157 were reported in 2017.^3^ Healthy farm animals and the farm environment can be sources of zoonotic STEC infections, which can be acquired in petting zoos or open farms where good hygiene may not always be rigorously enforced (Conrad et al., 2017). The consumption of contaminated food is also a source of infection (Treacy et al., 2019). Several virulence factors are associated with STEC, including Shiga toxin (*stx*) genes, which are key to the symptoms of the infection (Carter et al., 2008; Wu et al., 2008). High frequencies of resistance to antimicrobial agents in STEC, including O157 have also been reported in recovered patients (Mukherjee et al., 2017).

The study aimed to elucidate the transcriptional and adaptive changes (mutations) that can occur in *E. coli* O157:H7 to adapt to disinfectant exposure. We assessed the changes that occur with repeated exposure of *E. coli* O157:H7 to sub-lethal disinfectant concentrations from improper applications or from the presence of organic materials, such as faeces, which are common on farms and may decrease the efficacy of disinfectants, allowing *E. coli* O157:H7 to persist.

## Methods

### Strains and cultures

TUV93-0, a laboratory-adapted derivative of EDL933 (*E. coli* O157:H7) lacking the Shiga-toxin gene (*stxA1*), was used during this study (Campellone et al., 2002). For all studies, isolates were grown aerobically at 37°C on Luria-Bertani (LB) agar or broth as required.

### Disinfectants used in this study

Four disinfectants were selected, representing different chemical compositions commonly used on farms. Those selected contained the following active ingredients: peroxymonosulphate (PMS), quaternary ammonium compound (QAC) and glutaraldehyde, iodophor (IOD) and chlorocresol (CRE). In addition, a second disinfectant (QAC2), which contained quaternary ammonium compounds, was used for selected experiments. General Orders (GO) concentrations for each Defra-approved disinfectant are available.^4^ For products QAC, IOD, and CRE, a 4 x GO stock was prepared and a 2-fold dilution series was created to achieve 12 dilutions. The final concentration ranges were as follows: the QAC disinfectant ranged from 2.04% (v/v) to 0.001% (v/v), the CRE disinfectant from 2.17% (v/v) to 0.0011% (v/v) and the IOD disinfectant from 1.16% (v/v) to 0.0006% (v/v). For the PMS disinfectant, a 2x GO stock was prepared and a 2-fold dilution series was created to achieve 12 dilutions. The final concentration ranged from 0.5% (w/v) and 0.0002% (w/v). All disinfectants were diluted using WHO hard water (2 mM calcium chloride and 0.68 mM magnesium chloride).

### Determining inhibitory and sub-inhibitory disinfectant concentrations for TUV93-0

The survival and growth kinetics of TUV93-0 were determined using the FLUOstar (BMG, Labtech). TUV93-0 was grown for 16 h at 37°C in LB broth, then diluted (1,100) into fresh broth, with 150 μL of the diluted cells aliquoted into the wells of a 96-well plate. The Cells were grown for 150 min at 37°C in the FLUOstar, with absorbance (600 nm) measured at 15 min intervals. After 150 min, 50 μL of the diluted disinfectant or WHO water (control) was added, and the growth was monitored for a further 21.5 h. Each experiment was performed in triplicate, and the growth was compared to that of the cells grown in the presence of WHO water. The area under the curve (AUC) values were calculated for each growth curve using GraphPad Prism software.

### Repeated exposure of TUV93-0 to the sub-inhibitory disinfectants and the identification of antibiotic-resistant derivatives

TUV93-0 was grown overnight in LB broth at 37°C, then diluted (1:100) into fresh broth and grown for 2.5 h at 37°C with shaking (150 rpm) until the disinfectant was added at sub-inhibitory concentrations and grown for a further 21.5 h. The following day, the cultures were diluted (1:100), and the exposure was repeated every 24 h for a total of 72 h. After each passage, a sample of the cells was frozen at-80°C for future analysis.

The frozen stocks of *E. coli* exposed to the disinfectant were diluted in PBS to achieve 100–500 colonies/plate. Then, they were plated onto LB agar and grown for 16 h at 37°C. The colonies were replica plated onto LB-agar supplemented with either 8 mg/L nalidixic acid (8Nal) or 4 mg/L tetracycline (4Tet) using replicating cloths and incubated. The resulting colonies were counted, and some were selected for further analysis.

### Minimum inhibitory concentration

For the selected mutants obtained following repeated disinfectant exposure, Minimum inhibitory concentration (MIC) values were determined for ampicillin (Amp), tetracycline (Tet), chloramphenicol (Chl) and nalidixic acid (Nal) using agar dilution methods described elsewhere (Andrews, 2001). Control strains ATCC 25922 and NTCC 10418 were included along with TUV93-0. The breakpoints applied to the MIC values were as follows: Amp >8 mg/L, Tet >4 mg/L, Chl >8 mg/L and Nal >16 mg/L.

### Assessment of nalidixic acid resistance

The derivatives of TUV93-0 generated by disinfectant exposure and selected from 8Nal were assessed for mutations in *gyr*A, *par*C and *par*E. All three genes were amplified with previously described methods (Kim et al., 2009; Ling et al., 2003) and sequenced with ABI technology. The sequences were aligned using SeqMan Pro (DNAstar Lasergene), and single nucleotide polymorphisms (SNPs) were identified and compared to known mutations.

### Sequence analysis of the TUV93-0 derivatives

The selected isolates were whole genome sequenced (WGS) to identify the SNPs associated with antimicrobial resistance (AMR). DNA was extracted using the MagMAX CORE nucleic acid purification kit (ThermoFisher Scientific) and a KingFisher duo prime. The DNA was sequenced on the MiSeq platform (Illumina) following library preparation using Nextera XT. TUV93-0 was assembled with SPAdes 3.12 (Bankevich et al., 2012) and annotated using the *Escherichia-*specific database with Prokka 1.11 (Seemann, 2014) to produce a GenBank file. The mutants were mapped against the TUV93-0 GenBank file using Snippy 3.1 (Seemann, 2015) to identify the SNPs. Wherever indels or deletions were indicated by Snippy, the gene was translated to check for stop codons using the ExPASy translate tool.^5^ Valid SNPs or deletions were those with a depth of coverage >10 and 100% agreement for the mutated sequence across all reads.

### RNA-seq analysis of the disinfectant-exposed cells

RNA-sequencing (RNA-seq) was used to assess the effect of disinfectant exposure on gene expression. RNA was isolated from the TUV93-0 cells exposed to the PMS disinfectant and QAC disinfectant at final concentrations of 0.125% w/v and 0.002% v/v, respectively, for 24 h at 37°C. The RNA was stabilised using a solution of 95% Ethanol and 5% phenol for a minimum of 30 min and then frozen at-80°C until the RNA was extracted. The RNA was extracted from the pelleted cells using the SV total RNA isolation kit (Promega).

Ribosomal RNA was removed using the Ribo-Zero bacterial rRNA depletion kit (Illumina) with 2.5 μg of total RNA, following the manufacturer’s protocol. The remaining RNA was purified using magnetic beads and a modified RNAeasy MinElute clean-up method (Promega). The RNA was then converted to cDNA by reverse transcription using random hexamers (Roche or similar). The RNA samples were mixed with 400 μM of the random hexamers and incubated at 70°C for 10 min. The cDNA reaction mix contained AMV-reverse transcription buffer (1x), 100 mM DTT, 10 mM dNTPs, 12.5 U of Protector RNase inhibitor and 25 U of AMV reverse transcriptase and was incubated at 25°C for 10 min followed by 42°C for 60 min. The cDNA synthesis system (Roche or similar) was used to generate the second strand of the cDNA. The samples were purified using magnetic beads (AMPure, Agencourt) according to the manufacturer’s instructions before the ligation of the sequencing primer. The Nextera XT kit was used to generate the sequencing libraries before sequencing on the NextSeq (Illumina).

### Analysis of the RNA-seq results

Reads in the fastq files were trimmed using trimmomatric (Version 0.39; Bolger et al., 2014) with a sliding window of 10 bases, a quality cutoff of 20 and a minimum read length of 80. The reads were mapped to the *E.coli* O157:H7 EDL933 genome (accession number: AE005174) using the aligner Smalt (version 0.7.6; Institute S, 2014) and samtools (version 0.1.19; Li et al., 2009). FeatureCounts (Liao et al., 2014) was used to count the number of reads mapped to each gene, and the R package Deseq2 (Love et al., 2014) was used to carry out four different comparisons (Table 1) and generate a summary of the number of differentially expressed genes (DEGs; adjusted *p* < 0.05, Log2 Fold Change >2 and both filters combined). Volcano plots for the six contrasts are shown in Appendix 5. The gene ontology of the DEGs was determined using DAVID^6^ and Biocyc.^7^

**Table 1:** A summary of the number of the differential expression genes found from four comparisons involving TUV-93 and the adapted strains QAC4 and QAC8 (adjusted *p* < 0.05, Log2 Fold Change >1 or < −1 and both filters combined).

Comparison	Group A*	Group B*	Number of genes			
			Adjusted *p*-value (<0.05)	Log2 fold-change (> 1)	Log2 fold-change (< −1)	Both
1	TUV93_PMS	TUV93_Water	442	1,677	243	442
2	TUV93_QAC	TUV93_Water	12	29	37	12
3	TUV93_QAC	QAC4_QAC	48	78	351	48
4	TUV93_QAC	QAC8_QAC	20	64	31	20

*Group names include the strain tested (TUV93 and the adapted strains QAC4 and QAC8) and treatment (the PMS disinfectant, QAC disinfectant, and water).

## Results

### Defining the sub-lethal disinfectant concentrations

The sub-lethal concentration was determined for each disinfectant so that these levels could be used to assess transient and permanent genomic changes. For the O157:H7 strain TUV93-0, the lowest lethal concentrations were 0.5x (0.58% (v/v)) and 0.25x (0.25% (w/v)) GO strength for iodophor disinfectant (IOD) and peroxymonosulphate disinfectant (PMS), respectively. The chlorocresol-containing disinfectant (CRE) and glutaraldehyde and quaternary ammonium compound (QAC) inhibited the growth at much lower concentrations of 0.033x GO (0.067% (v/v)) and 0.002x GO (0.004% (v/v)), respectively. No growth inhibition was observed in the water control. Therefore, sub-lethal was defined as the highest concentration of the disinfectants at which growth was observed. Although the maximum OD 600 nm achieved after 24 h growth was lower for some disinfectants compared to the water control (Supplementary Data 1B).

### Transcriptome analysis of PMS disinfectant-exposed *Escherichia coli* TUV93-0

**Figure 1:**
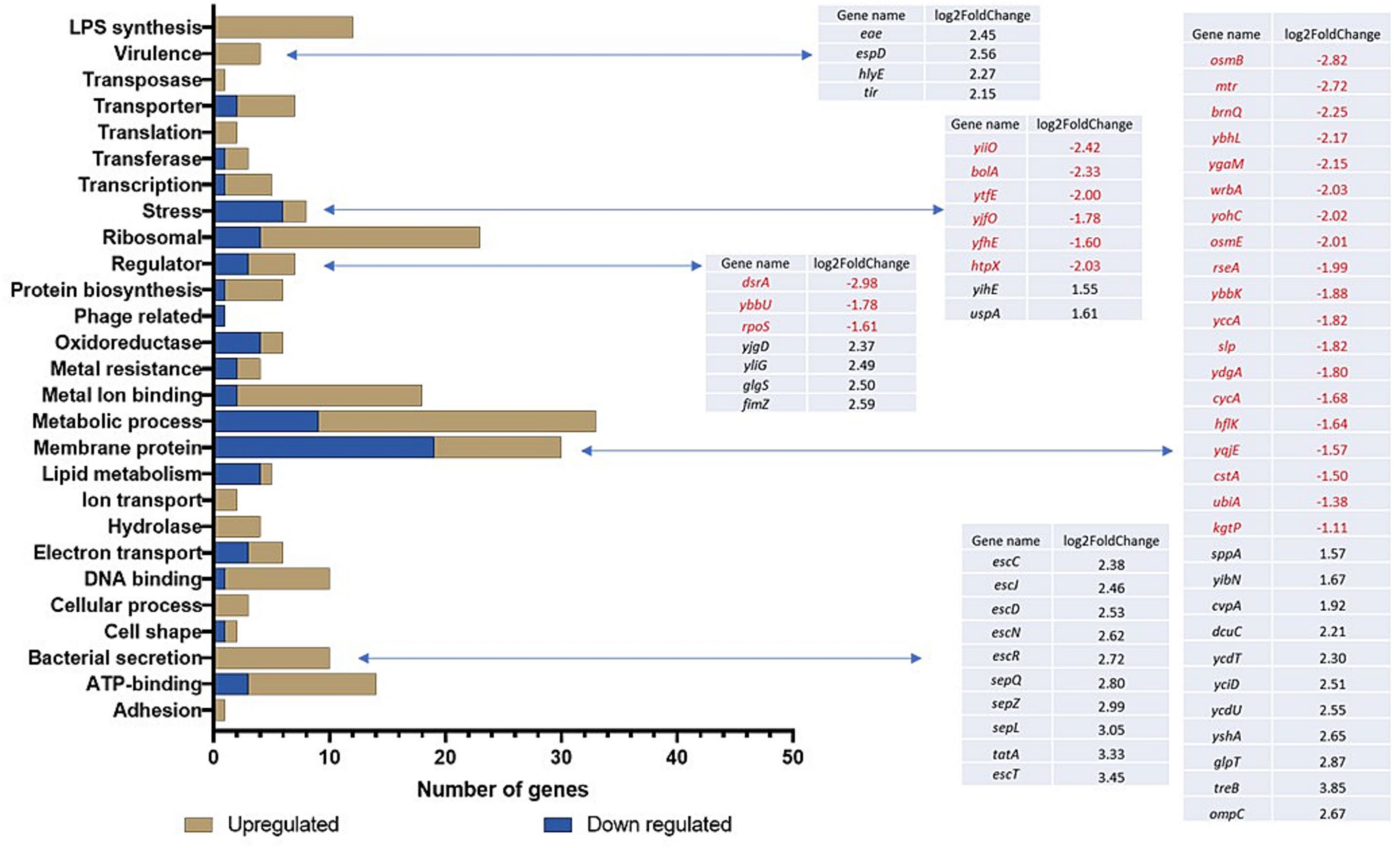
Gene ontology of the DEGs following exposure of TUV93-0 to the PMS-containing disinfectant. DAVID and BioCyc were used to define the categorisation of the genes.

Transcriptional changes were assessed following the exposure of TUV93-0 to the sub-lethal disinfectant concentrations using RNA-seq. In the presence of the PMS disinfectant, 442 genes were differentially expressed with a *p*-value <0.05 and log_2_ fold change >1 or < −1 (Table 1) when compared to the water control (Supplementary Data 1). The exposure of *E. coli* O157:H7 to the PMS disinfectant resulted in the upregulation of the expression of 341 genes. The differentially expressed genes (DEGs) were divided into functional groups using DAVID (Figure 1). The DEGs had a range of functions and included genes associated with transcription and translation, metabolic pathways, respiration and enzymes (Figure 1; Supplementary Data 2). The DEGs with the greatest fold change increase (>4 fold) were the ribosomal proteins (*rplC, rplW* and *rpsJ*) and several uncharacterised loci (Z5140, Z5881 and Z3062). A total of 215 genes (181 upregulated and 34 downregulated) were hypothetical proteins without functional classification.

Differential regulation of the genes associated with the bacterial membrane was observed (Figure 1) and included 12 genes associated with LPS synthesis (*wbdR, wbdO, wbdN, wbdQ, wbdP, waaD, wzy, waaI, waaL, waaY, waaJ* and *per*/*rfbE*), which were upregulated between 1.9 and 3.4-fold. In addition, one of the major outer membrane porins, *ompC*, known to be essential for the survival of *E. coli* in low pH conditions (Wang et al., 2021), was upregulated in response to the PMS disinfectant. Nineteen other membrane-associated genes were downregulated in TUV93-0 in response to the PMS disinfectant and included the osmotic shock proteins *osmB* and *osmE*, which maintain cell integrity and encode putative lipoproteins involved in the stress response (Brauer et al., 2023; Figure 1). Transporters can play a role in the response to disinfectant exposure as they facilitate transport across the cell membrane. Seven genes associated with transport across the cell membrane were differentially expressed, with *ygjU* and *argT* downregulated (1.2 to 1.8-fold) and *yaeT*, *yaeC*, *livJ*, *rbsB* and *malE* upregulated (1.3 to 2.6-fold; Supplementary Data 2). A stress response is associated with exposure to harsh environmental conditions, and eight stress-associated DEGs were identified in PMS-exposed TUV93-0 (Figure 1), six of which were surprisingly downregulated in the presence of the disinfectant.

Several virulence-associated genes of *E. coli* O157 were upregulated in response to the PMS disinfectant and included the type III secretion genes (*escC, escJ, escD, escN, escR, sepQ, sepZ, sepL, escT* and *espD*) and genes required for cell adherence to the gut epithelium (*tir, eae*; Figure 1). In addition, a 2-fold upregulation of the virulence-associated haemolysin E gene (*hlyE*) was observed.

### Transcriptome analysis of QAC disinfectant-exposed TUV93-0

Twelve DEGs were identified when TUV93-0 (*p* < 0.05 and log_2_ fold change >1 or < −1) was exposed to the QAC disinfectant and compared to the control (TUV93-0 exposed to water), four of which were downregulated and eight were upregulated under these conditions (Table 2). Five genes associated with the phage shock protein operon (*pspABCDE*) were upregulated >5-fold in the presence of the disinfectant. Two genes associated with heat shock were also upregulated (*mopB* and *htpX*), along with one hypothetical protein (*yjbO*). The genes downregulated during the disinfectant exposure included *gadA* and *gadC* (*xasA*), which are involved in response to acidic conditions, as well as *cyoC* and a putative transporter protein (Z3658).

**Table 2:** DEGs identified when TUV93-0 exposed to the QAC disinfectant was compared to TUV93 exposed to water (control).

Gene	Log2 fold-change	Adjusted *p*-value	Locus tag	Product annotation
*cyoC*	−1.36	0.0458	Z0533	cytochrome o ubiquinol oxidase subunit III
*xasA*	−2.24	0.0012	Z2216	acid sensitivity protein gadC putative transporter
*Z3658*	−1.59	0.0114	Z3658	putative transport system permease
*gadA*	−2.49	0.0196	Z4930	glutamate decarboxylase isozyme
*pspE*	5.23	1.17E-15	Z2477	phage shock protein
*pspD*	5.84	2.98E-15	Z2478	phage shock protein
*pspC*	5.47	1.04E-13	Z2479	phage shock protein, activates phage shock-protein expression
*pspB*	6.34	7.77E-19	Z2480	phage shock protein putative inner membrane protein
*pspA*	6.43	1.98E-22	Z2482	phage shock protein, putative inner membrane protein
*htpX*	1.59	0.0316	Z2876	heat shock protein, integral membrane protein
*yjbO*	5.21	1.78E-10	Z5648	hypothetical protein
*mopB*	2.05	0.0316	Z5747	GroE10 Kd chaperone binds to Hsp60 in pres. Mg-ATP suppressing its ATPase activity

### The effect of repeated exposure to the lowest sub-lethal concentrations of the disinfectant

Repeated exposure to sub-lethal concentrations of biocides has been shown to induce mutations in *Salmonella,* leading to antibiotic and biocide resistance. The repeated exposure of TUV93-0 to sub-lethal disinfectant levels was assessed for four disinfectants and water (control) for up to three passages. Approximately 3.2×10^4^ (1.67×10^4^ screened for tetracycline resistance and 1.59×10^4^ screened for nalidixic acid resistance) colonies were screened for resistance to antimicrobials acquired through chromosomal mutations for all disinfectants and controls. Twenty-five *E. coli* colonies (0.15% of the total colonies were screened for tetracycline resistance) generated from exposure to any disinfectant or control grew on 4Tet. Seven hundred and one *E. coli* colonies (4.4% of the total colonies screened for nalidixic acid resistance) generated from exposure to all disinfectants and controls grew on 8Nal (Supplementary Data 1A). Eighty-five per cent of the Nal^R^ colonies were generated following QAC exposure.

Nal MICs were determined for selected passaged isolates, with the levels increasing from 2 mg/L (TUV93-0 parent strain) to between 16 and 64 mg/L (Table 3). Sequencing of the topoisomerase genes (*gyA*, *gyrB*, *parC* and *parE*) in selected mutants, either through sequence analysis of PCR-generate amplicons (*gyrA*, *parC* and *parE*) or through whole genome sequencing (*gyrB*), identified several non-synonymous single nucleotide polymorphisms (SNP) in *gyrA* (G74, D82 and D87) and *gyrB* (K447; Table 4). The mutation of the amino acid D87 in *gyrA* was associated with the increased MIC of 64 mg/L. Mutations in the *gyrA* and *gyrB* genes had no effect on resistance to Amp, Tet and Chl.

**Table 3:** Antimicrobial susceptibility profiles of the TUV93-0 derivatives generated following disinfectant exposure at sub-inhibitory concentrations.

Strain	Disinfectant	Sub-culture	MIC (mg/l)			
			NAL	CHL	TET	AMP
TUV93-0	None	0	2	4	1	2
QAC4	QAC	2	8	**32**	**8**	2
QAC7	QAC	2	8	**16**	2	4
QAC8	QAC	2	16	4	2	4
QAC13	QAC	1	**64**	4	1	2
QAC21	QAC	1	**64**	4	0.5	2
QAC22	QAC	1	16	4	1	2
QAC33	QAC	2	8	**16**	2	2
QAC55	QAC	3	4	**32**	**8**	0.25
CRE35	CRE	1	**32**	4	0.5	2
PMS2	PMS	1	**32**	4	0.5	2
WHO11	Water	2	**64**	4	1	2

Values in bold are greater than breakpoint values.

**Table 4:** Mutations identified in the derivatives of TUV93-0 generated following disinfectant exposure at sub-inhibitory concentrations.

Strain	Gene	Start	Reference sequence	Mutant	Type of mutation	Consequence	AUC*
QAC4^#^	*marR*	91,827	CAAAAAAAC	CAAAAAC	Deletion	Frameshift_N126fs	6.32
	*toxB*	7,197	A	T	SNP	S2115S	
	Hypo protein 01831	147,311	TAAAAAAAT	TAAAAAAT	Deletion	Frameshift_F75fs	
QAC7^#^	*marR*	91,827	CAAAAAAAC	CAAAAAC	Deletion	Frameshift_N126fs	
	*toxB*	7,197	A	T	SNP	S2115S	
	Hypo protein 01831	147,311	TAAAAAAAT	TAAAAAAT	Deletion	Frameshift_F75fs	
QAC8^#^	*marR*	91,827	CAAAAAAAC	CAAAAAC	Deletion	Frameshift_N126fs	
	*toxB*	7,197	A	T	SNP	S2115S	
	Hypo protein 01831	147,311	TAAAAAAAT	TAAAAAAT	Deletion	Frameshift_F75fs	
QAC13^#^	*gyrA*		GAC	TAC	SNP	D87Y	
	*toxB*	7,197	A	T	SNP	S2115S	
	Hypo protein 01831	147,311	TAAAAAAAT	TAAAAAAT	Deletion	Frameshift_F75fs	
QAC21	*gyrA*		GAC	GGC	SNP	D87G	3.73
QAC22	*gyrA*		GGT	GCT	SNP	G74A	
QAC33^#^	*marR*	91,827	CAAAAAAAC	CAAAAAC	Deletion	Frameshift_N126fs	
	*toxB*	7,197	A	T	SNP	S2115S	
QAC55^#^	*Kup*	135,934	T	G	SNP	V228G	6.065
	*Ion*	115,176	T	G	SNP	D445E	
	*lacA*	29,771	G	A	SNP	P151L	
	*toxB*	7,197	A	T	SNP	S2115S	
	*marR*	91,781	GAA	GAAA	Insertion	Frameshift_Q110fs	
	Hypo protein 01831	147,311	TAAAAAAAT	TAAAAAAT	Deletion	Frameshift_F75fs	
CRE35	*gyrA*		GAC	AAC	SNP	D82N	1.281
PMS2^#^	*gyrB*	75,506	T	C	SNP	K447E	
WHO11	*gyrA*		GAC	GGC	SNP	D87G	3.78

*Calculated for the mutants grown in the presence of the QAC disinfectant at a concentration of 0.008% (v/v). The AUC value for TUV93-0 grown in the presence of 0.008% (v/v) QC disinfectant was 2.07.

# Isolates for which the WGS analysis was used to determine the presences of SNPs.

The derivatives of TUV93-0, generated following exposure to the sub-lethal QAC disinfectant, developed elevated Chl MIC (16–32 mg/L) and Tet MIC (2–8 mg/L; Table 3). This represented an increase between 3 and 4 doubling dilutions compared to TUV93-0. The analysis of the growth kinetics of QAC4 and QAC55 in the presence of the QAC disinfectant (Appendix 3) revealed that both isolates could grow in the disinfectant at a concentration of 0.004% (v/v), while TUV93-0 (parent strain) and QAC21 were unable to grow at this concentration. The isolates QAC4 and QAC55 were also able to grow in the presence of higher concentrations of a second QAC disinfectant (QAC2) compared to TUV93-0 or QAC21 (Supplementary Data 1B). The increase in tolerance to the disinfectant shown by QAC4 and QAC55 was not observed in the presence of the PMS disinfectant (data not shown).

The WGS analysis of QAC4, QAC7, QAC33 and QAC55 identified deletions and SNPs in the genes, including *marR* and *toxB* (Table 4). QAC4 and QAC7 also had identical SNPs in a hypothetical protein 01831. Frameshift deletions in *marR* and the gene encoding 01831 resulted in truncated proteins, while the changes in *toxB* were silent. In contrast to QAC55, *marR* carried an insertion that resulted in a frameshift, unlike the mutations observed in other derivatives. Mutations in the hypothetical protein 01831 and *toxB* were identical for all sequenced isolates. QAC55 carried three additional mutations not seen in the other isolates, which were non-synonymous SNPs in *kup, lon and lacA* (Table 4). Mutations in *marR*, *toxB* and the hypothetical protein 01831 were also observed in the derivatives of TUV93-0 with reduced susceptibility to Nal (QAC8 and QAC13; Table 4).

### Transcriptional profiling of the disinfectant-generated mutants

**Figure 2:**
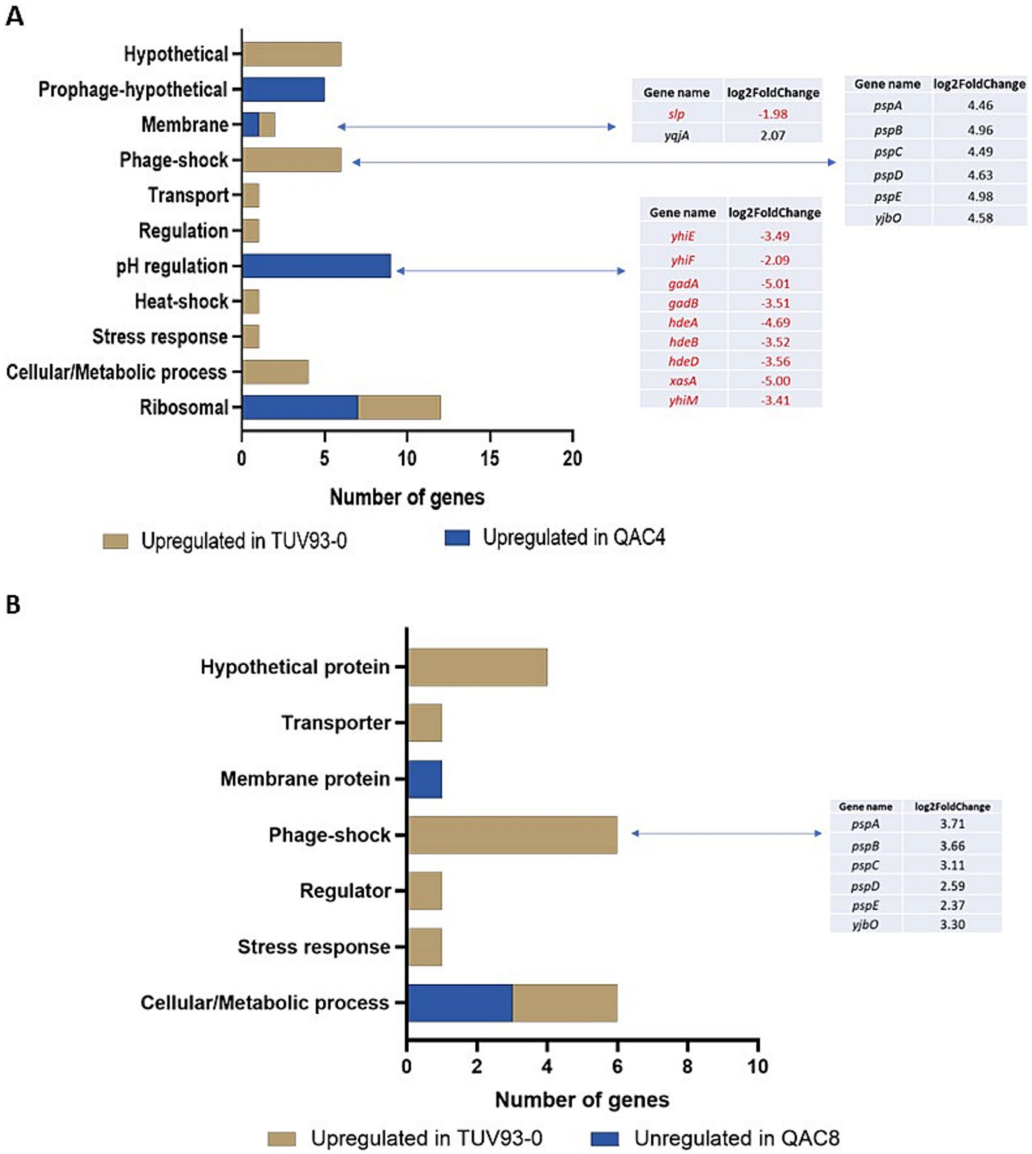
DEGs detected in the QAC4 (A) and QAC8 (B) mutants compared to TUV93-0 when exposed to the QAC-containing disinfectant. Genes highlighted in red were upregulated in QAC4 or QAC8, while genes highlighted in black were upregulated in TUV93-0 (parent).

Two isolates (QAC4 and QAC8) generated following passage in the QAC disinfectant were selected for transcriptome analysis using RNA-seq. These isolates were selected because QAC4exhibited a high MIC for Chl and Tet () and QAC8 exhibited an elevated MIC for Nal without topoisomerase mutations (). Twenty-six genes were upregulated in TUV93-0 compared to QAC4, while 22 genes were downregulated (Figure 2A; Supplementary Data 2). These genes were functionally classified using DAVID and Biocyc based on biological processes, wherever possible. Exposure of QAC4 to the QAC disinfectant led to the overexpression of ribosomal genes (*rplC, rplD, rplV, rpsC, rpsF, rpsJ and rpsS*), genes related to pH regulation and hypothetical prophage-related genes (Z1835, Z1842, Z1847, Z1849 and Z1854). There was a 3 to 5-fold upregulation in expression the pH response genes *gad*ABC, *gadE (yhiE)* and *hde*ABD in QAC4 (Figure 2A). In addition, the genes *yhiM, yhiF* and *slp,* known to be associated with glutamate-dependant acid resistance, were upregulated (1.9 to 3.4-fold) in QAC4 in response to the disinfectant.

The genes associated with metabolism (*ftsJ, glnA, glpD, miaA*), stress response (*htpX, htrA*) regulation (*mtlR*), transport (*mtr*), phage shock (*pspABCDE* and *yjbO*) and six hypothetical proteins were upregulated in TUV93-0 compared to QAC4 (Figure 2A). The phage shock-associated genes demonstrated a 5-fold increase in the expression within TUV93-0 (Figure 2A).

Sixteen genes were upregulated in TUV93-0 compared to QAC8, while four genes were downregulated in response to the QAC disinfectant (Figure 2B). Six genes associated with phage shock (*pspABCDE* and *yjb*O) were upregulated between 2-and 4-fold in TUV93-0 compared to QAC8. In addition, the genes associated with metabolism (*ynhG*, *glpD* and *cmk*), gene regulation (*mtlR*), transport (*ynfM*) and stress (*htpX*) were upregulated in TUV93-0 compared to QAC8. Four DEGs that encoded the hypothetical proteins (Z1638, Z1198, *ynh*G and *yej*G) were also upregulated in TUV93-0. The genes upregulated in QAC8 were involved in aerobic respiration (*sucB, sucD* and *nuoG*) and included a membrane protein (*wbdP*). A comparison of the adapted isolates in the presence of the disinfectant and water (control) was performed but not included in this study. For QAC4, no significant DEGs were detected between the QAC disinfectant-and water-treated cells, suggesting that the changes elicited by the disinfectant were unaffected by further disinfectant exposure. For QAC8, six DEGs were identified, including the *psp* genes.

## Discussion

The study aimed to determine how *E. coli* O157:H7, a zoonotic human pathogen, can survive and adapt to disinfection, which may aid its persistence on farms. Through transcriptomic and genomic analysis, many mechanisms were shown to be affected by disinfectant exposure in O157:H7. The transcriptional changes elicited by the disinfectants differed depending on their composition. The response observed in the presence of potassium peroxymonosulphate (PMS), an oxidising agent that kills bacteria by disrupting the bacterial membrane and causing cell wall rupture, was wider-ranging than that observed for the QAC-containing disinfectant. Merchel Piovesan Pereira et al. (2020) identified a significant variability in the response of *E. coli* to different disinfectants, which depended on the chemical composition of the product and the duration of exposure. They found that 35 genes were differentially expressed in response to benzalkonium chloride (BC) after 12 h of exposure and 396 genes after 30 min exposure, highlighting the variability in response over time. The authors also observed that there was a wide variability in the number of differentially expressed genes in the presence of different chemical compounds. Similarly, our study found distinct sets of DEGs depending on the disinfectant used.

The PMS-containing disinfectant elicited transcriptional changes in a wide range of genes associated with various cellular processes. One might expect differential expression of membrane-associated genes, transporter proteins and genes associated with stress response as they protect cell integrity from the deleterious effects of disinfectants. A number of these were identified. Porins play a role in the movement of chemicals, including antibiotics, across the cell membrane. Differential expression of *ompC* and *ompF* has been demonstrated in response to biocide exposure (Merchel Piovesan Pereira et al., 2021), where a decrease in expression was observed. In this study, an unexpected increase in the expression of *ompC* was observed, similar to the findings of Bore et al. (2007), who reported increased *ompC* expression in the presence of benzalkonium chloride (BC). This increase was linked to a switch to the expression of smaller porins. These differences may relate to the type of disinfectant tested. The LPS operon was significantly upregulated in the presence of PMS, and it has been shown that in *Proteus mirabilis,* overexpression of LPS-associated genes occurs upon cell exposure to chlorhexidine (Clarke et al., 2023). LPS is a protective mechanism for Gram-negative bacteria, helping to prevent the access of damaging substances (Bertani and Ruiz, 2018).

It is interesting to note that several genes associated with the locus of enterocyte effacement (LEE) region, which are associated with attachment to gut enterocytes and type III secretion, and *hlyE*, the pore-forming haemolysin, were upregulated in response to the PMS-containing disinfectant. This suggests that exposure to this disinfectant enhances the expression of virulence-associated genes. The regulatory genes *rpoS* and *dsrA* were differentially expressed in the presence of the PMS disinfectant and are known regulators of LEE expression (Gaytán et al., 2016; Franzin and Sircili, 2015; Laaberki et al., 2006). However, in this study, the expression of *rpoS* and *dsrA* were downregulated in the presence of the disinfectant, while the LEE genes were upregulated. The expression of the *hlyE gene* may be related to glucose or oxygen starvation, both of which are known to increase its expression (Wyborn et al., 2004). It is unclear why the expression of the virulence gene might be upregulated in response to PMS exposure. It is known that LEE gene expression can be influenced by environmental factors, including acidity, antibiotics (e.g., ciprofloxacin; Kijewski et al., 2024) and quorum sensing by gut metabolites, which also plays a key role in LEE expression (Gelalcha et al., 2022). In future studies, the effect of disinfectant exposure on small RNAs should be considered as they are known to play a role in the stress response of O157 (Segura et al., 2021).

The QAC-containing disinfectant was found to induce the expression of the *psp* operon, which is expressed in *E. coli* at the stationary phase and in response to stressful environmental conditions (Li et al., 2009). These genes have not been previously associated with disinfectant exposure but are highly expressed in mature biofilms (Beloin et al., 2004) and are essential for ensuring the correct location of secretin in the cell membrane (Srivastava et al., 2017). BC, a constituent ingredient of the QAC disinfectant, is known to damage the bacterial cell membrane, leading to cell death (Maris, 1995). The expression of *psp* genes during exposure to BC, as shown here, may therefore counteract the deleterious effect of these compounds, representing a novel role for this operon that has not previously been described.

Transcriptome analysis of the disinfectant-adapted isolates found that the genes involved in the regulation of pH were upregulated in response to disinfectant exposure. These included *hdeABD*, *gadA* and *gadE* (*yhiE*), which are co-localised within the “acid fitness island” of O157 (Carter et al., 2012), as well as *slp* (Li and Morigen, 2022), *yhiF* and *yhiM. YhiF* and *yhiM* have been associated with acid resistance and the negative regulation of LEE gene expression (Masuda and Church, 2003; Tree et al., 2011). *GadB* and *gadC* (*xasA*), which are also components of the acid resistance system, are localised elsewhere (Tramonti et al., 2017). In addition, the study of Forbes et al. examined the effect of repeated exposure to BC on *E. coli* gene expression, in which they identified many DEGs with a range of functions (Forbes et al., 2019). Merchel Piovesan Pereira et al. (2020) also found that the upregulation of acid stress genes (*hde* and *gad*) was observed during disinfectant exposure, including during exposure to glutaraldehyde-containing products. Glutaraldehyde is a constituent of the QAC disinfectant used in this study. These mechanisms may aid in the survival of QAC4 in disinfectants, but it is unlikely that they will contribute to the observed elevation in tolerance to antibiotics.

Disinfectant-adapted isolates have been generated in *E. coli* (Merchel Piovesan Pereira et al., 2021; Bore et al., 2007) and *Salmonella* (Whitehead et al., 2011; Webber et al., 2015) in several studies. These studies found a role for *marA* and *acrA* in the development of antibiotic resistance to quinolones, chloramphenicol and ampicillin, as well as in biocide tolerance, including triclosan. Exposure to sub-lethal concentrations of several disinfectants induced point mutations in various genes within *E. coli* O157:H7. The mutations in *lon,* which are known to stabilise and increase levels of *marA*, in combination with the mutation of *marR*, which also increases the expression of *marA*, can result in resistance to chloramphenicol and tetracycline (Nicoloff et al., 2007). The mutation of *lon* has not previously been associated with disinfectant exposure, and although our results suggest an effect, the role of *lon* mutations on resistance and *marA* expression requires future work. None of the isolates generated by repeated exposure to the disinfectants displayed an MDR phenotype, which one might expect when MAR is involved. However, the level of *mar*A overexpression is also believed to influence the phenotype (Pourahmad Jaktaji and Ebadi, 2013), which may explain the phenotypic differences observed in this study.

In addition to resistance to chloramphenicol and tetracycline, the isolates exposed to the disinfectant containing QAC, which also included BC, readily developed resistance to nalidixic acid. Spontaneous mutations in *gyr*A can occur through repeated passage of *E. coli*. However, the rate at which the mutations occurred in the presence of the QAC disinfectant was accelerated with respect to the water control. Mutations in *gyr*A and *gyr*B are commonly linked to nalidixic acid resistance. The mutation at D87 led to the largest increase in MIC. Previous studies have shown that BC exposure in *Salmonella typhimurium* can generate nalidixic acid-resistant mutants (Guo et al., 2014).

Also for future consideration is the role of mutations in *mar*R N126, a region associated with protein dimerization, which has been linked with levofloxacin resistance (Bhatnagar and Wong, 2019). Although resistance to levofloxacin was not tested in this study, mutants with this change in *marR* were identified in our study, showing an elevated nalidixic acid MIC.

Therefore, this study demonstrated that in the presence of sub-lethal disinfectant levels, bacteria can adapt to survive. Crucially, we noted that even short-term exposure to some disinfectants was sufficient to elicit these changes both in the genome and transcriptome of *E. coli* O157, highlighting the importance of accurately following recommendations for applying and maintaining lethal disinfectant concentrations on farms. We speculate that these changes occur not only in *E. coli* O157:H7 but possibly in other pathogenic *E. coli* strains as well, enabling them to persist in the environment and making them less sensitive to disinfectants. The generation of antibiotic-resistant isolates through biocide exposure may have broader implications as it has been shown that nalidixic acid-resistant *E. coli* with topoisomerase mutations are more likely to be multidrug-resistant (AbuOun et al., 2020), and QAC exposure can promote the conjugal transfer of plasmids (Han et al., 2019). Therefore, in sub-optimal disinfectant concentrations, *E. coli* O157 may not only survive and persist but may also experience an increase in AMR through mutations and increased plasmid transfer, which could impact the AMR burden. Although this study primarily focused on the resistance of pathogenic *E. coli* to common farm disinfectants, we believe these findings could also apply to other settings, such as hospitals and homes, where disinfection is used to control bacterial pathogens.

### Data summary

All sequence data from this study are deposited in the European Nucleotide Archive (ENA) within project PRJEB34679. The mutants generated by exposure to disinfectant are stored under the following accession numbers: SAMEA7566848, SAMEA7566847, SAMEA7566852, SAMEA7566851, SAMEA7566846, SAMEA7566850 and SAMEA7566849. The data from the transcriptome analysis are stored under the following sample accession numbers: SAMEA5986189, SAMEA5986181, SAMEA5986175, SAMEA7566633, SAMEA5986184, SAMEA5986177, SAMEA5986173, SAMEA7566628, SAMEA7566631, SAMEA5986182, SAMEA7566624, SAMEA7566626, SAMEA5986179, SAMEA5986186, SAMEA7566629, SAMEA7566630, SAMEA7566632, SAMEA7566622, SAMEA7566623, SAMEA7566625 and SAMEA7566627.

## Data Availability

The datasets presented in this study can be found in online repositories. The names of the repository/repositories and accession number(s) can be found at: https://www.ebi.ac.uk/ena, PRJEB34679.

## Footnotes

^1^ https://www.gov.uk/guidance/defra-approved-disinfectant-when-and-how-to-use-it

^2^ https://www.cdc.gov/infectioncontrol/guidelines/disinfection/introduction.html

^3^ https://assets.publishing.service.gov.uk/government/uploads/system/uploads/attachment_data/file/774291/STEC_O157_report.pdf

^4^ http://disinfectants.defra.gov.uk/DisinfectantsExternal/Default.aspx?Module=ApprovalsList_SI

^5^ https://web.expasy.org/translate/

^6^ david.ncifcrf.gov

^7^ biocyc.org/ECOLI/class-tree?object=Gene-Ontology-Terms
