# Supplementary material for: Use of transcriptomics and genomics to assess the effect of disinfectant exposure on the survival and resistance of Escherichia coli O157:H7, a human pathogen
Source: Front Microbiol. 2024 Oct 23;15:1477683. doi: 10.3389/fmicb.2024.1477683 (PMC11538004; doi:10.3389/fmicb.2024.1477683)
Supplement: Supplementary file 1 [file Table_1.DOCX]

**Supplementary data 1**

**A.**  Number of colonies tested to identify derivatives of TUV93-0 able to grow in the presence of 4Tet or 8Nal generated by repeated disinfectant exposure at sub-inhibitory concentrations.

B. Growth kinetics of mutants of TUV93-0 generated after exposure to sub-inhibitory concentrations of product B.

C. Volcano plots for the 4 comparisons.

**A. Number of colonies tested to identify derivatives of TUV93-0 able to grow in the presence of 4Tet or 8Nal generated by repeated disinfectant exposure at sub-inhibitory concentrations.**

| **Disinfectant** | **Disinfectant conc. (%w/v or v/v)** | **Tetracycline** | | **Nalidixic acid** | |
| --- | --- | --- | --- | --- | --- |
|  |  | **Colonies Tested** | **Presumptive TetR (%)** | **Colonies Tested** | **Presumptive NalR (%)** |
| PMS (A) | 0.125 | 5057 | 0 (0) | 5420 | 37 (0.68) |
| QAC (B) | 0.002 | 2831 | 23 (0.8) | 2287 | 597 (26.1) |
| Product C | 0.145 | 1459 | 1 (0.068) | 1243 | 10 (0.80) |
| Product D | 0.034 | 3174 | 1 (0.031) | 3212 | 31 (0.96) |
| WHO standard water | N/A | 4183 | 0 (0) | 3800 | 26 (0.68) |

**B. Growth kinetics of mutants of TUV93-0 generated after exposure to sub-inhibitory concentrations of QAC disinfectant.**

**A:** Isolate QAC4, QAC21, and QAC55, and TUV93-0 were grown in the presence of 0.008% (v/v) product B (final concentration) supplemented LB broth and **B.** in the presence of product E supplemented into LB broth at a final concentration of 0.009% (v/v). Error bars indicate the standard deviation for replicate testing. Graphs were generated using GraphPad Prism 7.

**A**

**B**

**C. Volcano plots for the 4 comparisons.** The two vertical and one horizontal dashed lines corresponds to 2-fold changes and 0.05 alpha (for the adjusted p-values). (Dis A is PMS disinfectant and Dis B is QAC disinfectant)

| 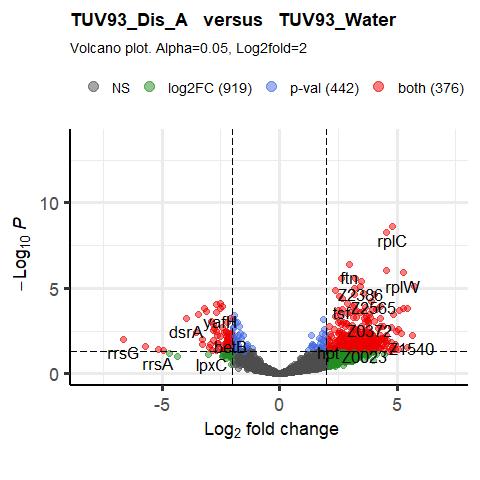 | 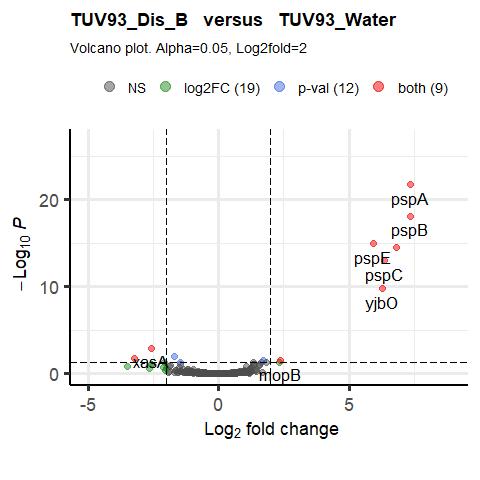 |
| --- | --- |
| 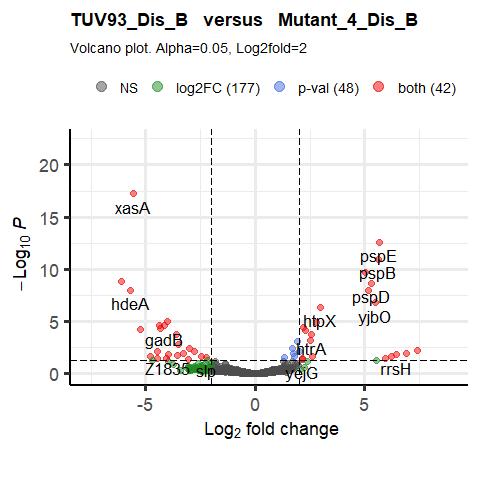 | 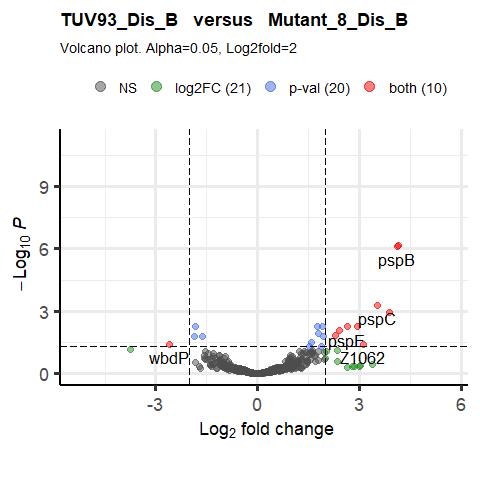 |
